# Clinical outcomes of an early intervention program for preschool children with Autism Spectrum Disorder in a community group setting

**DOI:** 10.1186/1471-2431-13-3

**Published:** 2013-01-07

**Authors:** Valsamma Eapen, Rudi Črnčec, Amelia Walter

**Affiliations:** 1 Academic Unit of Child Psychiatry, South West Sydney Local Health District (AUCS), Sydney, Australia; 2 University of New South Wales, Sydney, Australia

**Keywords:** Autism, Behavioral intervention, Early intervention, Cognitive function, Developmental outcomes

## Abstract

**Background:**

Available evidence indicates that early intervention programs, such as the Early Start Denver Model (ESDM), can positively affect key outcomes for children with Autism Spectrum Disorder (ASD). However, programs involving resource intensive one-to-one clinical intervention are not readily available or deliverable in the community, resulting in many children with ASD missing out on evidence-based intervention during their early and most critical preschool years. This study evaluated the effectiveness of the ESDM for preschool-aged children with ASD using a predominantly group-based intervention in a community child care setting.

**Methods:**

Participants were 26 children (21 male) with ASD with a mean age of 49.6 months. The ESDM, a comprehensive early intervention program that integrates applied behaviour analysis with developmental and relationship-based approaches, was delivered by trained therapists during the child’s attendance at a child care centre for preschool-aged children with ASD. Children received 15–20 hours of group-based, and one hour of one-to-one, ESDM intervention per week. The average intervention period was ten months. Outcome measures were administered pre- and post-intervention, and comprised a developmental assessment - the Mullen Scales of Early Learning (MSEL); and two parent-report questionnaires - the Social Communication Questionnaire (SCQ) and Vineland Adaptive Behaviours Scales–Second Edition (VABS-II).

**Results:**

Statistically significant post-intervention improvements were found in children’s performance on the visual reception, receptive language and expressive language domains of the MSEL in addition to their overall intellectual functioning, as assessed by standardised developmental quotients. Parents reported significant increases in their child’s receptive communication and motor skills on the VABS-II, and a significant decrease in autism-specific features on the SCQ. These effects were of around medium size, and appeared to be in excess of what may have been expected due to maturation. Nonetheless, these results need to be confirmed in a controlled study.

**Conclusions:**

This study suggests community dissemination of the ESDM using predominantly group-based intervention may be an effective intervention. Making the ESDM accessible to the wider ASD community in child care settings has the potential for significant clinical and economic benefits. Further studies are indicated in this area, including those with younger children, and which incorporate a control group and standardised ASD assessments.

**Trial registration:**

This trial is registered with the Australian New Zealand Clinical Trials Registry: Registry number ACTRN12612000461897.

## Background

Autism Spectrum Disorder (ASD) is a life-long neurodevelopmental disorder characterised by impairments in social interaction, verbal and non-verbal communication, and a restricted repertoire of activities and interests [1]. The prevalence of ASD is rising worldwide [2], with ASD estimated to affect around 1 in every 100 persons [3].

ASD is a disorder of significant public health importance that confers substantial personal, social and economic disadvantage. Building on genetic vulnerability [4], it has been hypothesised that ASD emerges from a developmental cascade in which a deficit in attention to social stimuli leads to impaired interactions with primary caregivers. This results in abnormal development of the neurocircuitry responsible for social cognition, which in turn adversely affects later behavioural and functional domains dependent on these early processes, such as language development [5]. Such a model suggests the importance of early intervention for ASD, and is supported by studies showing better outcomes with earlier treatment [6,7]. Moreover, early intervention in the first years of life offers the best potential for children as brain plasticity is greatest during this period, enabling the establishment and reorganisation of neuronal networks in response to environmental stimulation [5].

Several meta-analyses conducted in recent years have tended to conclude that Early Intensive Behavioural Intervention (EIBI), incorporating the principles of applied behaviour analysis (ABA), is the treatment of choice for young children with ASD (cf. [8,9]). The literature indicates that superior outcomes are associated with entry into EIBI at the earliest possible age [10,11]. Consistent with this view, the American Academy of Pediatrics has recommended that children be screened for ASD from around 18 months of age to aid in early intervention efforts [12]. However, the only comprehensive EIBI program available for children aged less than 30 months that has been empirically evaluated is the Early Start Denver Model (ESDM: [13]). The ESDM is a manualised, comprehensive play-based intervention that integrates ABA with developmental and relationship-based approaches [14]. While evidence for the efficacy of the ESDM has been found among children aged less than 30 months [13], the ESDM is designed for children with ASD aged from 12 months to preschool age, and further research on its effectiveness among preschool-aged children is warranted.

The first and only randomised controlled trial of the ESDM demonstrated significant gains in visual processing and improvements in language abilities, with subsequent gains in IQ and adaptive behaviours, among children receiving the ESDM [13]. In that study, the intervention was provided by trained therapists and children received 20 hours per week of one-to-one ESDM intervention in a University clinic setting. There was also a separate parent training module.

It is important to investigate the effectiveness of other less resource intense methods of delivery of the ESDM. An alternative would be to offer this intervention to preschool-aged children in group settings and to reduce the parent training component, which would allow wider access to this evidence-based intervention. Given the play-based nature of the ESDM, this intervention may be particularly well-suited to the preschool setting. A recent study by Vivanti et al. of 21 preschool-aged children (mean age 38 months) sought to determine predictors of treatment response following one year of group-based ESDM [15] where the staff-to-child ratio was 1:3. Vivanti et al. reported significant developmental gains following the intervention, with skills in functional use of objects, goal understanding and imitation predicting positive response to treatment [15].

The ESDM is based on the Denver Model, an earlier variant with a greater focus upon play-based learning. Preliminary data are already available for the efficacy of the Denver model [16,17] with reports of positive outcomes in cognitive, social, language and play skills, as well as a reduction in ASD symptomatology, in children with ASD. The Rogers and Lewis [16] study of 31 children (mean age 45 months), which employed a staff-to-child ratio of 1:2 with children treated in groups of six using the Denver Model, found significant improvement in play skills and reduction in autism symptoms.

Studies exploring the effectiveness of EIBI interventions, such as ABA, in community settings have produced generally favourable results [18], although effect sizes reported are typically smaller than in clinic-based efficacy studies. Some of these effectiveness studies have explored EIBI in preschool settings, although these have generally adopted a one-to-one approach (e.g., [19-23]). One exception was Magiati et al. [19] who reported a two-year follow-up of 16 preschoolers treated with an eclectic range of EIBIs in autism specific nurseries with staff-to-child ratios ranging from 1:1 to 1:3.3. While children improved in their mental age scores in that study, IQ standard scores were unaffected after treatment.

The present study is therefore amongst the first to report on the effectiveness of the ESDM intervention for preschool-aged children in a community-based group setting.

## Methods

### Ethical approval

The study had the approval of the Human Research Ethics Committees of the South Western Sydney Local Health District, and also from the University of New South Wales. All families recruited to the study provided informed consent to participate, and the research was conducted in accordance with the ethical standards outlined in the Helsinki Declaration.

### Design

A pre-post study of children treated with group ESDM was conducted.

### Participants

Participants were 26 children with a DSM-IV-TR diagnosis of Autistic Disorder, made by a community-based physician, who were attending an Autism Specific Early Learning and Care Centre (ASELCC) in metropolitan Sydney, Australia. The centre is one of six ASELCCs established by the Australian Government within the setting of a long day child care centre for children aged two-to-six years.

Exclusion criteria applied were known neurodevelopmental (e.g., Fragile X Syndrome) or neurological (e.g., epilepsy) disorders, and significant vision, hearing, motor or physical problems.

The average age of children at the time of study commencement was 49.6 months (SD 6.08, range: 36-to-58 months) and 21 (81%) were male. The average age of the participating children’s mothers was 39.2 years (SD 5.2, range: 28-to-52 years). English was the primary language spoken at home in 84% of families, although 63% of families reported a cultural background other that Australian. Twenty-two percent of primary caregivers had completed postgraduate education, 52% tertiary, 22% secondary, and 4% primary only. None of the participants were receiving an EIBI outside of the ESDM intervention offered as part of the study. No families withdrew from the study during the course of the intervention; however, there were some instances of missing data due to families not completing measures within the necessary timeframes.

### Intervention

This study employed the previously published ESDM curriculum and teaching principles [14] within a group setting. The ESDM involves play-based, active engagement of the child across many aspects of their development, with priorities on functional communication, social interaction, cognition, play, and positive behaviours. Other than accommodations to allow translation to the group context, no modifications were made to the ESDM curriculum.

Participants received two half-hour intensive individualised ESDM therapy sessions per week (i.e., 1 hour per week), in addition to 15-to-20 hours of ESDM group intervention, during their attendance at the centre. There were two rooms in the centre, each with up to ten children. The staff-to-child ratio was 1:4. The one-to-one sessions were conducted by a consistent key worker. There was no specific parent training component to the intervention, however optional parent education evenings were offered at the centre.

All interventions were delivered by therapists formally trained in the ESDM by accredited trainers. Each child had an individualised treatment plan that incorporated a range of objectives, dependent on the child’s level of functioning. These objectives were taken from the ESDM curriculum, which includes a list of skills across four levels in a range of domains. These domains included receptive communication, expressive communication, social skills, joint attention behaviors, fine motor, gross motor, imitation, cognition, play skills, behavior and personal independence (eating, dressing, grooming, chores). Examples of items from the curriculum checklist for the receptive communication domain include “looks to partner when name is called” (level 1), “identifies by pointing or showing several named body parts on self or other person” (level 2), “differentiates at least four different colours upon request” (level 3), and “understands comparatives: bigger, shorter, smaller, most, least, few, many, etc.” (level 4). A curriculum checklist was completed for each child at the commencement of the intervention to establish the child’s current level of abilities in each of these domains across the four levels, and the next more advanced skills that were not yet a regular part of the child’s repertoire became treatment objectives for their one-to-one intervention.

In terms of the group component of the ESDM, the preschool setting and learning activities were designed to entice small groups of children such that each child’s objectives could be targeted in structured group learning experiences. This required the clinician to create joint activity routines that were child-led and which would attract the child’s attention and create learning opportunities using ESDM-based instruction. For example, during natural play routines, the therapist became a play partner and children were encouraged to model and imitate, trade materials back and forth between themselves and with the therapist, name or point to objects, actions and relations. During group activities, children were expected to communicate intent using words or gesture or both, and turn taking was facilitated using sounds and words as well as body language such as eye contact, gestures, movements and actions. Particular challenges that needed attention within the group setting included managing child attention by reducing competing stimuli, supporting communication by using both verbal and visual-auditory cues, supporting temporal sequencing, and managing unwanted behaviours.

### Measures

The **Mullen Scales of Early Learning** (MSEL: [24]) is a widely used, standardised measure of early development for children aged from birth to 68 months, yielding standardised *T* Scores and age equivalent scores on the following subscales: Visual Reception, Fine Motor, Receptive Language, Expressive Language, and Gross Motor*.* The Gross Motor subscale was not administered in this study.

Given the majority of children in the current sample did not receive MSEL subscale raw scores that were high enough for calculation of a meaningful *T* score (i.e., they were performing at a level <0.1 percentile), standardised developmental quotients (DQs) were calculated for each subscale of the MSEL by dividing each child’s age equivalent score by their chronological age at the time of testing and multiplying by 100, as is common practice. In this regard, a child who was aged 48 months, but who had an age equivalent score of 24 months, would receive a DQ of (24/48) × 100 = 50. Given the DQ is standardised according to the child’s age, a child with a stable developmental trajectory would achieve approximately the same DQ at two time points despite their age equivalent scores increasing [e.g., (18/36) × 100=50 at 36 months (three years) of age; and (24/48) × 100 = 50 at 48 months (four years) of age etc.]. Conversely, a child whose developmental trajectory had shifted could achieve a higher or lower DQ over time [e.g., (18/36) × 100 =50 at three years of age; and (42/48) × 100 =88 at four years of age]. Thus, comparing change in DQ score over time may help to control for the effects of development and maturation.

An overall DQ was also calculated for each child by taking the average of the child’s DQs for the four completed subscales in order to provide an estimate of overall intellectual ability. Note that the sum of the *T* scores for these four subscales (i.e., Visual Reception, Fine Motor, Receptive Language and Expressive Language) is used to calculate the Early Learning Composite Score of the MSEL. It should also be noted that the DQs calculated in this study are not equivalent to *T* scores or the Early Learning Composite Score of the MSEL, but represent an attempt by the study team to standardise scores for the purpose of making comparisons over time.

Parents of participating children completed two measures.The **Social Communication Questionnaire** (SCQ: [25]) is a 40-item measure of autism-specific symptoms where scores of 15 or more indicate probable ASD. The SCQ has robust psychometric properties [26-28]. The **Vineland Adaptive Behaviour Scales Second Edition** (Parent Form) (VABS-II: [29]) assesses parents’ perceptions of their child’s everyday adaptive functioning in the domains of Communication (including expressive and receptive language), Daily Living Skills, Socialisation and Motor Skills. For each domain, including an overall Adaptive Behaviour Composite, a norm-referenced standardised score with a mean of 100 and SD of 15 is calculated. V-scale scores with a mean of 15 and a SD of 3 are calculated for each sub-domain. The VABS-II has well-established strong psychometric properties [29].

All measures were administered at two time points (on entry to the program, and again on exit or 12 months after entry, whichever occurred first) by a member of the research team. Parents also completed a demographic questionnaire at the start of the study.

### Statistical analyses

The results of the previous ESDM trial [13], which used the same dependent measures as utilised in the current design, indicate large effect sizes. Despite changes to the intervention modality in the current study, clinically meaningful effects of medium effect size (specifically, of Cohen’s *d*=0.6 or above) were predicted. In this regard, a sample of 26 was sufficient to provide experimental power >0.8, with alpha at 0.05 using a pre-post design.

Paired samples t-tests were conducted to compare children’s scores on the MSEL, SCQ and VABS-II pre- and post-intervention. Analyses were conducted using SPSS statistical software. Alpha was set at 0.05 for all comparisons, following recommendations by Saville [30], who argues for this per-comparison level rather than a family-wise approach when conducting research in novel areas. Cohen’s *d* effect sizes were also reported. Following the recommendations of Dunlap et al. [31], and in order to provide a conservative estimate of the size of observed effects, Cohen’s *d* scores were calculated using the pooled standard deviation uncorrected for the correlation between pre- and post-scores. Dunlap et al. argued that when pre-post scores are highly correlated, as was the case in this study (average correlation for variables reported was *r =* .79), correction for the correlation results in a significant over-estimate of the true effect size [31]. It is widely accepted that Cohen’s *d* values of 0.2 – 0.49 denote small sized effects; 0.5 – 0.79 denote medium sized effects; and >0.8 denote large effect sizes.

## Results

The average time between initial and follow-up assessment was 9.72 months (SD 2.91).

### Mullen scales of early learning

**Figure 1:**
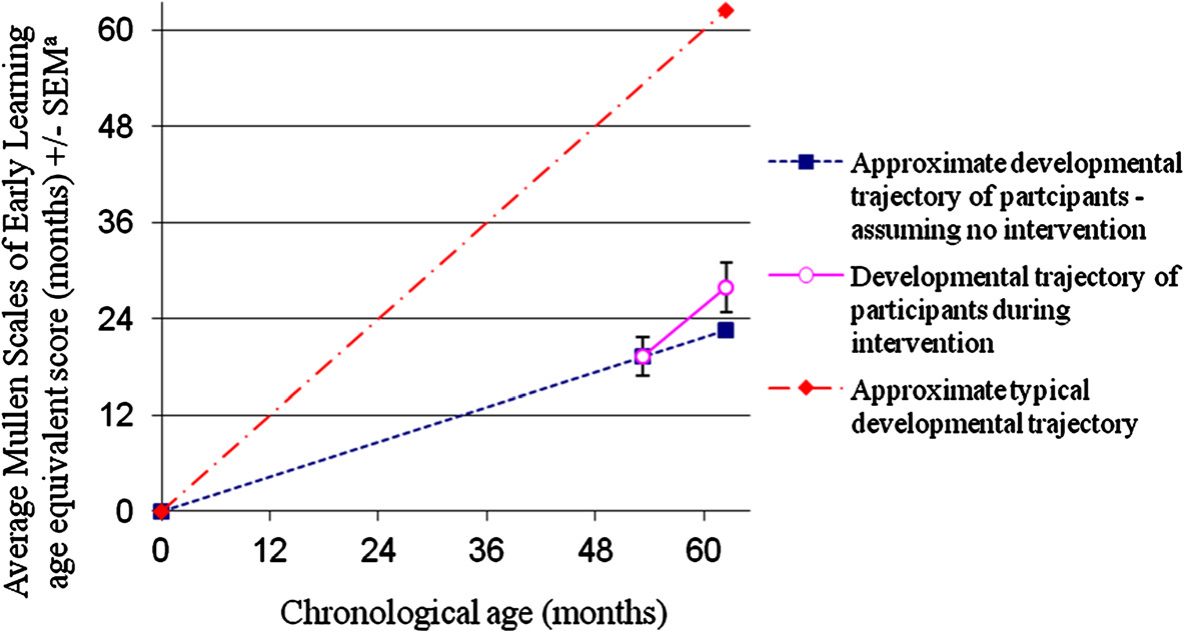
**Approximate developmental trajectory of study participants before and during group ESDM intervention, compared to a typical developmental trajectory.** a. MSEL scores were calculated by taking the average of the age equivalent scores obtained in the four completed MSEL subscales (i.e., Visual Reception, Fine Motor, Receptive Language and Expressive Language) in order to provide an estimate of overall intellectual ability.

Results of paired samples t-tests and Cohen’s *d* effect sizes are shown in Table 1. There was a significant increase in children’s mean overall DQ, a standardised index of their overall intellectual functioning, from pre- to-post-intervention. The size of this effect was Cohen’s *d* = .47, which is approaching medium size. The change in approximate developmental trajectory suggested by these results is depicted in Figure 1. Significant improvement was also found in children’s performance on the Visual Reception, Receptive Language and Expressive Language scales of the MSEL, as assessed by standardised DQs. No significant increase was found in children’s DQ on the Fine Motor scale.

**Table 1:** Pre- to post-intervention scores in a cohort of preschoolers treated with group ESDM

	**Time 1**		**Time 2**					
	**Mean**	**SD**	**Mean**	**SD**	***t***	**df**	***p***	**Cohen’s *d***^**f**^
**Mullen Scales of Early learning**								
Visual Reception DQ^a^	35.9	20.6	51.5	28.2	−3.9	19	0.001**	0.63
Fine Motor DQ^a^	47.9	26.1	51.3	21.6	−1.1	20	0.28	0.14
Receptive Language DQ^a^	27.6	21.5	38.7	24.2	−4.4	18	<0.001**	0.48
Expressive Language DQ^a^	32.6	18.7	40.7	21.8	−4.0	20	0.001**	0.40
Overall MSEL DQ^b^	36.8	19.7	46.7	22.6	−5.6	18	<0.001**	0.47
**Vineland Adaptive Behaviour Scales-II Scale scores**								
Receptive Communication^c^	7.6	3.5	9.3	3.4	−3.2	15	0.005**	0.49
Expressive Communication^c^	7.6	2.8	7.8	2.8	−0.5	17	0.64	0.07
Written Communication^c^	11.7	2.4	11.9	3.7	−0.3	17	0.76	0.06
Personal Daily Living Skills^c^	8.2	2.5	8.2	2.0	−0.2	16	0.88	0.00
Domestic Daily Living Skills^c^	10.0	2.1	10.6	3.3	−1.0	17	0.34	0.22
Community Daily Living Skills^c^	9.8	2.7	9.2	3.4	1.3	17	0.20	0.20
Interpersonal Relationships^c^	8.1	2.5	8.7	3.0	−1.2	16	0.24	0.22
Play and Leisure Time^c^	7.9	2.0	7.5	2.1	1.3	15	0.20	−0.20^g^
Coping Skills^c^	11.4	3.1	11.5	2.9	−0.2	15	0.84	0.03
Gross Motor Skills^c^	9.3	2.8	11.7	2.7	−4.9	16	<0.001**	0.87
Fine Motor Skills^c^	9.3	3.9	9.7	2.8	−0.9	15	0.40	0.12
**Vineland Adaptive Behaviour Scales -II Standard Domain Scores**								
Communication^d^	61.6	14.1	65.3	20.1	−1.1	15	0.30	0.21
Socialisation^d^	66.8	13.0	67.1	14.5	−0.2	15	0.88	0.02
Daily Living Skills^d^	64.2	14.1	64.9	16.7	−0.3	16	0.75	0.05
Motor Skills^d^	66.4	17.8	73.7	14.2	−2.7	15	0.02*	0.45
Adaptive Behaviour Composite^d^	61.9	13.9	63.6	14.4	−0.9	14	0.38	0.12
**SCQ Total Score**^e^	18.5	7.2	15.7	7.1	2.2	18	0.04*	−0.39^g^

Note: * p<.05, ** p<0.01, SCQ = Social Communication Questionnaire.

a. DQ = (age equivalent score/chronological age) × 100.

b. Overall MSEL DQ = (Visual Reception DQ + Fine Motor DQ+ Receptive Language DQ + Expressive Language DQ)/4.

c. V-Scale score (mean: 15, SD: 3).

d. Standard score (mean: 100, SD: 15).

e. Range = 0–40. Scores of 15 or more denote probable ASD.

f. Following the recommendations of Dunlap et al. [31] Cohen’s *d* sores were calculated using the pooled standard deviation score uncorrected for the correlation between pre-post scores.

g. Negative effect sizes denote a reduction from pre-to-post. For the SCQ Total Score, lower scores are indicative of fewer ASD symptoms.

### Vineland adaptive behaviour scales - 2^nd^ edition

Significant increases were found in children’s standard scores on the Receptive Communication and Gross Motor sub-domains, in addition to the Motor Skills domain, of the VABS-II from pre- to post-intervention (see Table 1).

### Social communication questionnaire

There was a significant decrease in children’s total SCQ scores, indicative of a decrease in autism-specific features, from pre- to post-intervention (see Table 1). The percentage of children who scored below the clinical cut-off of 15 or more on the SCQ doubled from 21% at Time 1 to 42% at Time 2. In addition, the percentage of children who were reported to be using full sentences, as assessed by a specific item on the SCQ, increased from 52% at Time 1 to 68% at Time 2.

It is to be noted these are pre-post results that did not involve comparison with a control group.

## Discussion

The findings from the present study suggest that preschool-aged children with ASD receiving the ESDM intervention in a group format showed statistically and clinically significant gains on a range of clinical outcomes, particularly in the areas of receptive language and communication.

Given that the ESDM is designed to enhance the social attention and communicative abilities of young children with an ASD, with particular focus on the critical skills of social attention, affect sharing, imitation and joint attention, the significant gains made in the respective domains of visual reception and receptive and expressive communication are noteworthy. Charman et al. found that language outcomes were positively associated with early joint attention [32]. Thus, joint attention, imitation and intentional communication emerge as important characteristics to target in early intervention programs to optimise outcomes. Further, the play-based program offers opportunities for motor development, and our findings of improvement in the Motor Skills domain of the VABS-II is testament to this. There were also significant reductions in autism-specific features, as reported by children’s parents.

Due to the lack of a control group in the present study, it is difficult to establish a direct link between the observed improvements and the ESDM intervention. While it could be argued that the observed gains, or a substantial proportion of the observed gains, were due to maturational or other effects, we would contend that this is improbable for several reasons. As suggested by Perry et al. [23], such a conclusion is not consistent with what we know from the literature on outcomes for children with Autism in the past and as reported by several investigators in their control groups. For example, in a review of 23 longitudinal studies of IQ stability in children with ASD, all of whom were receiving therapy, Begovac et al. reported that the clear majority of studies found IQ to be stable over time [33]. Similarly, Kleinman et al. [34] did not report notable changes in MSEL scores in a sample of 46 children with Autistic disorder who were first assessed between 16 and 35 months and again between 42 and 82 months, an age range analogous to that used in the present study. While it is the case that some studies have found that a proportion of children with ASD show catch-up intellectual development during the preschool years that may not be attributable to intervention effects [35], it appears that this occurs more in children who have less severe Autism symptoms, a diagnosis of PDD-NOS, and higher IQ initially. Indeed, for children with more severe presentations, regression is a common course [33]. Thus, extrapolating from the current literature and given the fact that the participants in the present study had Autistic Disorder, high levels of ASD symptoms and low baseline IQ scores (<0.1 percentile), it appears that the cognitive improvements observed from pre- to post-testing are unlikely to arise as a result of maturation. In this regard, the significant cognitive improvements found among children in the present sample are promising, and suggest treatment, rather than maturation, effects. Future research with a control group is necessary to confirm this hypothesis.

Similarly, we would contend that the changes to SCQ and VABS-II scores are attributable to treatment rather than maturational effects for two main reasons: 1) Without intervention, ASD symptoms are generally thought to be stable over time, particularly where symptoms are at the more severe end of the spectrum [34]; and 2) The SCQ and VABS-II have acceptable test-retest reliability, suggesting that measurement errors were not a key factor.

In addition to contributing to earlier evidence about the benefits of the ESDM as an efficacious early intervention for toddlers with ASD, our findings also indicate that the ESDM may be effective when delivered in a group setting with preschool-aged children, with a relatively minimal one-to-one component, a finding consistent with the results of Vivanti et al. [15]. This finding has significant clinical implications in terms of wider dissemination opportunities for this evidence-based intervention. The findings of the present study are promising, given not only that the children received a less intensive intervention than that provided by Dawson and colleagues [13], but also because the children in the current study were older than those in the Dawson et al. sample at the commencement of the intervention (mean age of 49.6 months compared to 23.9 months), and had more severe developmental delay (MSEL composite scores <0.1 percentile compared to approximately 0.5 percentile). Despite this, effect sizes detected in this study, while lower, compare favourably with those achieved by Dawson et al. for the similar comparison of baseline-to-one-year outcome data in their ESDM group [13]. For example, the Cohen’s *d* effect size for overall MSEL developmental quotient was 0.47 in the current study, as compared to 0.87 in Dawson et al. [13]. Note that the effect sizes for the Dawson et al. study have been calculated by the authors of this study using the means and standard deviations for the ESDM group baseline and one-year outcome data reported by Dawson et al. [13]. See Table 2 for all available effect size comparisons.

**Table 2:** **Effect sizes observed in the current study compared with those observed in Dawson et al.**[13]

	**Cohen’s *d *observed in current study**	**Cohen’s *d *observed in Dawson et al. study**^**a**^[13]
**Mullen Scales of Early Learning**^**b**^		
Visual Reception	0.63	0.40
Fine Motor	0.14	−0.10 ^c^
Receptive Language	0.48	1.56
Expressive Language	0.40	1.03
Overall MSEL	0.47	0.87
**Vineland Adaptive Behaviour Scales-II Standard Domain Scores**		
Communication	0.21	0.52
Socialisation	0.02	−0.43^b^
Daily Living Skills	0.05	−2.15^b^
Motor Skills	0.45	0.38
Adaptive Behaviour Composite	0.07	−0.49^b^

a) Note that the effect sizes for the Dawson et al. study have been calculated by the authors of this study using the means and standard deviations for the ESDM group baseline and one-year outcome data reported in the Dawson et al. manuscript. Effects sizes were not corrected for the correlation between pre-post scores following the recommendations of Dunlap et al. [31].

b) In the present study, MSEL scale and overall scores were calculated using DQs, as described in the methods. In Dawson et al. [12] these were calculated using *T* scores and the Overall MSEL score was the Early Learning Composite Score.

c) Negative effect sizes denote a reduction from pre-to-post.

Overall, our findings weren't as strong as Dawson et al.’s, and this is in keeping with the existing evidence that one-to-one intervention starting at the youngest possible age offers the best opportunity for improving outcomes in children with ASD. However, our findings suggest that there are benefits to a less intensive intervention with older children in resource constrained environments. While the development of children in the present study remained well below age-based expectations after intervention, there was evidence that some ground had been recovered over the course of the intervention that may suggest a change in developmental trajectory.

Further, the fact that the intervention was offered in the context of a long day child care centre equivalent to a 'real world' environment is encouraging. Intervention programs provided within day care and preschool settings offer greater opportunities for generalisation of skills and improvements to school readiness, resulting in easier transition to other education settings following intervention.

Limitations of this study, such as the lack of a control group and lack of a standardised observational measure of ASD diagnosis and severity, need to be kept in mind while interpreting the significance of these results, and further research addressing these issues is warranted. In addition, studies involving group intervention with younger children and follow-up studies to ascertain whether treatment effects are durable are indicated.

ASD is a life-long disorder, having a major impact on quality of life, both for the individuals affected and their families, and producing a disproportionate burden on the public health and education systems. Furthermore, 75% of those with ASD also have associated intellectual disability, further compromising educational and future vocational opportunities. While there is no known cure, the most promising avenue to improve outcomes and avert this disease burden is early intervention. The findings of this study offer promise for centre-based early intervention programs that can be both cost-effective and accessible to the wider ASD community.

## Conclusions

The present project, an initial study of a community dissemination of the ESDM early intervention for ASD within child care settings, has the potential for significant clinical and economic benefits. Our findings of significant clinical improvements from the delivery of the ESDM in a less costly, more sustainable, group-based child care program provide a strong suggestion of the feasibility and effectiveness of this empirically validated treatment approach for children with ASD in the community during their critical early years of development.

## Abbreviations

ASD: Autism spectrum disorder; ABA: Applied behaviour analysis; ESDM: Early start denver model; EIBI: Early intensive behavioural intervention; ASELCC: Autism specific early learning and care centre; MSEL: Mullen scales of early learning; SCQ: Social communication questionnaire; VABS-II: Vineland adaptive behaviour scales second edition; DQ: Developmental quotient.

## Competing interests

The authors have no competing interests to declare.

## Authors’ contributions

VE made substantial contributions to conception and design of the study and interpretation of data as well as being involved in drafting and finalising the manuscript. RC was involved in study planning, data analysis and interpretation, and in manuscript preparation. AW contributed to data collection and analysis and to drafting the manuscript. All authors read and approved the final version of the manuscript for publication.

## Authors’ information

VE is Professor and Chair of Infant, Child and Adolescent Psychiatry at University of New South Wales and Head of the Academic Unit of Child Psychiatry South Western Sydney local Health District (AUCS). RC is a conjoint senior lecturer at the University of New South Wales School of Psychiatry and a practising child and adolescent clinical psychologist. AW is a Research Associate at the University of New South Wales, and a practising clinical psychologist.

## Pre-publication history

The pre-publication history for this paper can be accessed here:

http://www.biomedcentral.com/1471-2431/13/3/prepub
